# New Records of the Andean Cat (*Leopardus jacobita*) in the Central Andes of Chile: Filling Gaps in the Distribution Range through Private-Social Partnerships

**DOI:** 10.3390/ani12050639

**Published:** 2022-03-03

**Authors:** María Belén Zapararte, Constanza Napolitano, Martín Sapaj-Aguilera, Tomás Dinges, Catherine Kenrick, Gabriel Llerena-Reátegui, Cintia Gisele Tellaeche, Rocío Palacios

**Affiliations:** 1 Parque Andino Juncal, Los Andes 2100000, Chile; martinsapaj@gmail.com (M.S.-A.); tdinges@gmail.com (T.D.); catherine.kenrick@gmail.com (C.K.); 2 Departamento de Ciencias Biológicas y Biodiversidad, Universidad de Los Lagos, Osorno 5311157, Chile; 3 Andean Cat Alliance (Alianza Gato Andino, AGA), Villa Carlos Paz 5152, Argentina; gallerejs@gmail.com (G.L.-R.); cintiatellaeche@gmail.com (C.G.T.); rociopalacios@gmail.com (R.P.); 4 Institute of Ecology and Biodiversity (IEB), Concepción 4070374, Chile; 5 Cape Horn International Center (CHIC), Puerto Williams 6350000, Chile; 6 Asociación para la Conservación de la Biodiversidad PRO CARNIVOROS, Arequipa 04001, Peru; 7 Museo de Historia Natural de la Universidad Nacional de San Agustín de Arequipa, Arequipa 04001, Peru; 8 CETAS (Centro de Estudios Territoriales Ambientales y Sociales), Universidad Nacional de Jujuy, Jujuy 4600, Argentina

**Keywords:** Andean cat, *Leopardus jacobita*, central Andes of Chile, endangered, partnerships

## Abstract

**Simple Summary:**

The Andean cat is an endangered medium-sized felid with grey fur and a characteristic long tail with black rings. It is found only in cold and arid regions of the high Andes of Argentina, Bolivia, Chile, and Peru. As part of a collaboration between the Andean Cat Alliance (AGA) and Parque Andino Juncal (PAJ), we conducted a monitoring program from December 2020 to May 2021 using eight trail cameras in PAJ in the central Andes of Chile, an area where few records of the species have been described. We obtained records of Andean cats which fill a gap on the species distribution map, specifically in the area located between two previously identified evolutionary significant units (ESU) which are populations with different evolutionary histories that should be monitored, managed, and protected separately. Based on this new information, a comprehensive ESU reassessment across the range is currently being conducted by AGA. This will allow for a better understanding of the number of distinct populations across the range and the connectivity (i.e., current gene flow) among them, enabling the prioritization of small and isolated populations or units (i.e., higher extinction risk) and recommendations for evidence-based conservation strategies across the distribution.

**Abstract:**

The Andean cat (*Leopardus jacobita*) is one of the most endangered and least known wild cat species in the Americas and the world in general. We describe new records of the Andean cat in the central Andes of Chile, in Parque Andino Juncal, obtained as part of a monitoring program conducted from December 2020 to May 2021 using eight trail cameras. The cameras were active for 135 days (sampling effort 1080 camera-trap days). We recorded Andean cats in two different cameras, corresponding to two independent events in January and March 2021, respectively (0.19% capture success). Our new records are relevant since they fill a gap on the species distribution map, specifically in the area located between the two previously identified evolutionarily significant units (ESU) (26–35° S) which has been prioritized by the Andean Cat Alliance (AGA). We highlight the relevance of private protected areas in ecosystems of high biodiversity and fragility such as Parque Andino Juncal and also of strategic private-social partnerships for successful collaborative efforts to monitor the presence of rare, elusive, and endangered species. Our next steps will be to collect scats from this newly identified site and conduct genetic analyses to determine whether these animals are part of previously identified ESUs or a yet unidentified potentially distinct ESU or MU requiring special conservation measures.

The Andean cat (*Leopardus jacobita*) is one of the most endangered and least known wild cat species in the Americas and the world in general [1]. Its distribution range is restricted to arid regions of the high Andes of Argentina, Bolivia, Chile, Peru, and also a portion of the northern Patagonian steppe in Argentina [2,3,4] (Figure 1). Climatic conditions associated with the species presence and habitat specialization are extreme, with low temperatures, low precipitation, and large diurnal thermal variations [1].

The Andean cat is a medium-sized felid, weighing 4.0–5.8 kg [1,5] with mainly ash grey fur and brown-yellowish blotches distributed as vertical lines at both sides of the body (giving the appearance of continuous stripes). Its tail is characteristic; it is very long (66–75% of the head and body length), fluffy, thick and cylindrical, with six to nine wide rings of a dark brown to black color [1]. The Andean cat is thought to be a solitary species, but may be seen in pairs during the mating season or with kittens [6].

The species is classified as ‘endangered’ by the IUCN, with the number of mature individuals estimated to be 1378 and a decreasing population trend having been noted [1]. The main threats to Andean cats are habitat fragmentation and degradation, particularly that caused by extractive industries such as mining and oil, and other impacts to water sources, reduction of prey populations, and hunting [4]. Transmission of pathogens from domestic animals have been assessed as a potential threat, with no confirmation of its risk to date [7].

Andean cat populations occur in low densities, ranging from 0.018 ind/km^2^ (1.8 Andean cats in 100 km^2^) (central western Bolivia; [8]) to 0.07–0.12 ind/km^2^ (7–12 Andean cats in 100 km^2^) (northwestern Argentina; [9]). These populations have a patchy distribution due to a specialization for naturally fragmented rocky habitats [1] where its main prey, the viscacha (*Lagidium viscacia*) resides [10].

Probably associated with low densities and small population sizes, Andean cat populations have extremely low genetic diversity [11]. An analysis of the genetic structure of the populations across the range identified two ESU (different evolutionary history between units; [12]), with a latitudinal separation between 26° and 35° S [11]. Within the northern ESU, two genetically distinct groups are considered separate management units (MU, demographically independent populations with low connectivity between units; [12]) [11] (Figure 1). Interestingly and unlike the other populations or units, the southern ESU is found in low-altitude habitats (i.e., 800 masl) [1]. These units are operationally significant target groups for conservation (that is, populations that should be monitored, managed and protected separately, therefore guiding strategic conservation planning across the range).

Since 1999, the Andean Cat Alliance (AGA, by its initials in Spanish for Alianza Gato Andino), has conducted research and conservation initiatives across the species’ range. AGA’s ‘In the Field 24/7’ program aims to fill the gaps in the distribution range and identify conservation units across the range (i.e., ESU, MU or other units defined for conservation purposes). The area located between the two identified ESU (26–35° S) is currently an information gap for the Andean cat, and has been prioritized by AGA to conduct field assessments. Within this prioritized area, records of Andean cats are scarce [1,3].

Parque Andino Juncal (PAJ) is a private protected area (i.e., lands owned, administered, financed and operated by a private entity for conservation purposes) located in the central Andes of Chile, in the Aconcagua Valley (Valparaíso region; 32°54′57.49″ S 70°05′34.42″ W). PAJ encompasses 13,796 hectares, with its eastern limit the border with Argentina (Figure 1). The park has a Mediterranean climate, and includes shrublands, grasslands, rocky areas, wetlands, and glaciers, harboring vegetation and fauna typical of the high-altitude Andean ecosystem. In 2010, the park was categorized as a Ramsar site (Ramsar Convention on Wetlands of International Importance), a global priority area for the conservation of wetlands. The park represents an oasis of conservation surrounded by industrial projects such as mining and hydroelectric energy activities. Due to its geographic location, high elevation (from 2200 to more than 5000 masl) and Andean ecosystem, PAJ is an ideal hotspot for the potential presence of the Andean cat, therefore representing an area of major interest for Andean cat monitoring.

AGA fosters strategic partnerships with crucial local conservation actors working in the field, including private, governmental, and social organizations for mutual strengthening and to increase the success of monitoring efforts in prioritized areas. In this context, in 2020 a successful AGA-PAJ private-social partnership was established for a collaborative effort to monitor the potential presence of the Andean cat in PAJ. Private-social partnerships are methods of co-operation between private entities (in this case, PAJ) and social society organizations (in this case, AGA, a conservation organization).

**Figure 1:**
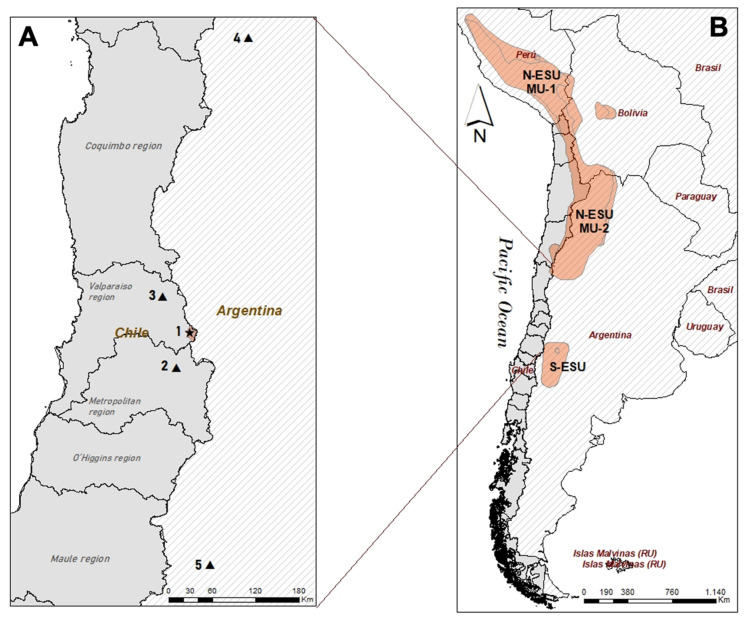
Location of the new Andean cat records in Parque Andino Juncal (PAJ), in the central Andes of Chile. (**A**) Central Chile and central-western Argentina. New record in Parque Andino Juncal (PAJ) indicated by (star). 1. Parque Andino Juncal, Chile boundaries are shown in brown. Previously recorded most adjacent Andean cat records (triangles) are shown for context: 2. Valle Nevado, Chile [13]; 3. Putaendo Valley, Chile [14]; 4. San Guillermo National Park, San Juan, Argentina (within Northern ESU, [6]); 5. Caverna de las Brujas, Mendoza, Argentina (within Southern ESU, [2]). (**B**) Known distribution of the Andean cat is shown in brown [1]. Andean cat identified Northern ESU (N-ESU) with its two distinct management units (MU-1 and MU-2) and Southern ESU (S-ESU) are shown. New Andean cat records in PAJ are located in the information gap area between ESU.

Here we report the first records of Andean cats in Parque Andino Juncal using trail cameras, obtained as part of a monitoring program conducted from December 2020 to May 2021 (to avoid cold season when cameras may be covered by snow due to high altitude). Eight unbaited trail cameras (Bushnell Trophy Cam, Bushnell Corporation, Overland Park, KS, USA) were installed within the park limits, at approximately 1 km apart. The cameras were active for 135 days (sampling effort 1080 trail camera days).

We recorded Andean cats in two different cameras, corresponding to two independent events (considered as independent events when images of the same species were >60 min apart) (0.19% capture success; captures/trail camera-days). One Andean cat photo was recorded in a trail camera located on a rocky cliff at 3052 masl (10,013 ft) in January 2021 (6:53 a.m.), near a wetland and an active viscacha den and colony. This trail camera also recorded puma, birds, viscacha, small rodents and reptiles during the same monitoring period. Six Andean cat photos (minutes apart, same event) were also recorded on another trail camera located on a wildlife path at 2933 masl (9622 ft) in March 2021 (03:29 a.m.), near the same wetland, and active viscacha den and colony (Figure 2A). This trail camera also recorded birds, guanaco, puma, hare and viscacha during the same monitoring period.

When this monitoring program ended, PAJ kept one trail camera from June to October 2021 in the same previous site located on a rocky cliff at 3052 masl where one Andean cat photo had been recorded. This camera was active for 126 days (sampling effort 126 trail camera days) and recorded Andean cats in five independent events (4% capture success) (one event in July 2021, 3:00 a.m.; four events in August 2021, 8:52 a.m., 12:49 p.m., 18:53 p.m., 20:07 p.m.) (Figure 2B–D).

Considering all of the Andean cat obtained pictures in the two trapping sessions, only for some of the records we could assess the animals’ coat pattern at least at one side to allow individual identification, suggesting those records belong to a single individual (Figure 2B,D).

Our new records are relevant since they fill a gap on the species distribution map, and add to four previous Andean cat reports in the area. In the central Andes of Chile, a record in Valle Nevado [13] (Figure 1 and Figure 2) and another in the Putaendo Valley [14] (Figure 1, site 3) have been described. In central-western Argentina, a record in San Guillermo National Park, San Juan [6] (Figure 1, site 4) and another in Caverna de las Brujas, Mendoza [2] (Figure 1, site 5) have been previously described and are the closest confirmed records in this area.

Our records also confirm the presence of the endangered Andean cat in Parque Andino Juncal, being the first record in a private protected area in the Valparaíso region in Chile, and highlighting the high ecological value of this conservation area. The central Andes of Chile is an ecosystem of high biodiversity and fragility. Among its main threats are extensive livestock rearing, mining and industrial development [15]. PAJ has extreme climatological conditions characterized by intense cold, snow, drought and high radiation and comprises a hydric network including rivers, glaciers, wetlands and underground springs. An important diversity of fauna and flora, endemic species as well as endangered species, a large number of migratory birds and higher vertebrates, are represented in the area, some of which are prey for Andean cats (viscacha, other small and medium-sized rodents, and birds; [10]), and which in general promote a healthy and stable ecosystem and associated ecosystem services. Conservation practices in Parque Andino Juncal are strict insofar as the only activities allowed are associated to recreation, education and scientific research. Industrial activities are not allowed.

The relevance of the central Andes of Chile for Andean cats is crucial. Very few records have been described in this area (this study, [13,14]), which poses a question on potential lower Andean cat densities compared to other areas across the range [8,9]. This area is also the southern-most limit of Andean cat distribution in Chile [1]. Also, higher human population densities and extractive activities take place in this area, posing potential higher threats for these unique populations [14,15]. Finally, the central Andes of Chile, along with the adjacent central-western Argentina area are located between the described Northern and Southern ESU, which suggests that a more in-depth study of Andean cat populations in this area and its potential connectivity with the two ESU is necessary.

Our next steps will be to collect scats from this newly identified site and conduct genetic analyses [10,11] to determine whether these animals are part of previously identified ESUs or a yet unidentified potentially distinct ESU or MU requiring special conservation measures. In the light of these and other recent new Andean cat records, a comprehensive ESU/MU reassessment across the range is currently being conducted by AGA. Implications of the results of genetic analyses will allow a better understanding of the number of distinct populations or units across the range and the connectivity (i.e., current gene flow) among them. Updated ESU and MU information will enable the prioritization of small and isolated populations or units (i.e., higher extinction risk) which, along with the assessment of local threats, will permit the recommendation of evidence-based conservation strategies across the distribution.

## Figures and Tables

**Figure 2:**
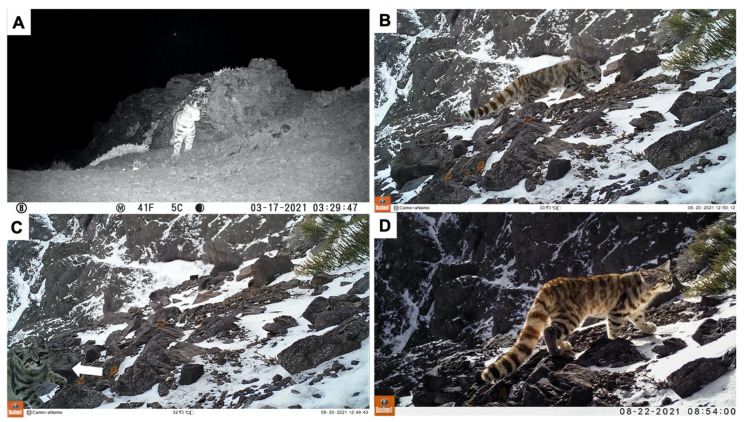
Andean cat photos obtained in two different sites in Parque Andino Juncal, from March to August 2021. (**A**) Trail camera located on a wildlife path at 2933 masl. This trail camera captured six Andean cat photos (minutes apart, same event) in March 2021. (**B**–**D**) Trail camera located on a rocky cliff at 3052 masl. This trail camera captured six independent events in January, July and August 2021.

## Data Availability

The data presented in this study are available on request from the corresponding author. The data are available upon request with restrictions from Andean Cat Alliance database.
